# Essential Oil Disinfectant Efficacy Against SARS-CoV-2 Microbial Surrogates

**DOI:** 10.3389/fpubh.2021.783832

**Published:** 2021-12-14

**Authors:** Emily S. Bailey, Marina Curcic, Jnev Biros, Hüseyin Erdogmuş, Nurcan Bac, Albert Sacco

**Affiliations:** ^1^Julia Jones Matthews Department of Public Health, Texas Tech University Health Sciences Center, Abilene, TX, United States; ^2^Edward E. Whitacre Jr. College of Engineering, Texas Tech University, Lubbock, TX, United States; ^3^EPS Fragrances, Istanbul, Turkey

**Keywords:** coronavirus, SARS-CoV-2, essential oil, surrogate, surface

## Abstract

Reports of COVID-19 cases potentially attributed to fomite transmission led to the extensive use of various disinfectants to control viral spread. Alternative disinfectants, such as essential oils, have emerged as a potential antimicrobial. Four essential oil blends were tested on three different surfaces inoculated with a coronavirus surrogate, bacteriophage Phi 6, and a bacterial indicator, *Staphylococcus aureus*. Log_10_ concentration reductions were analyzed using GraphPad Prism software. Data collected in this study show that the application of dilute essential oil disinfectants using a spray delivery device is an effective way to reduce concentrations of bacterial and viral microorganisms on ceramic, stainless steel, and laminate surfaces. Surrogate viruses were reduced up to 6 log_10_ PFU and bacterial were reduced up to 4 log_10_ CFU. Although surfaces are no longer considered a high risk fomite for COVID-19 transmission, the disinfection of microorganisms on surfaces remains an important consideration for high touch areas in hospitals, waiting rooms, etc. The application of spray disinfectants, based on essential oil blends, provides a rapid and effective means to reduce microbial contamination on high-touched surfaces.

**Graphical Abstract:**
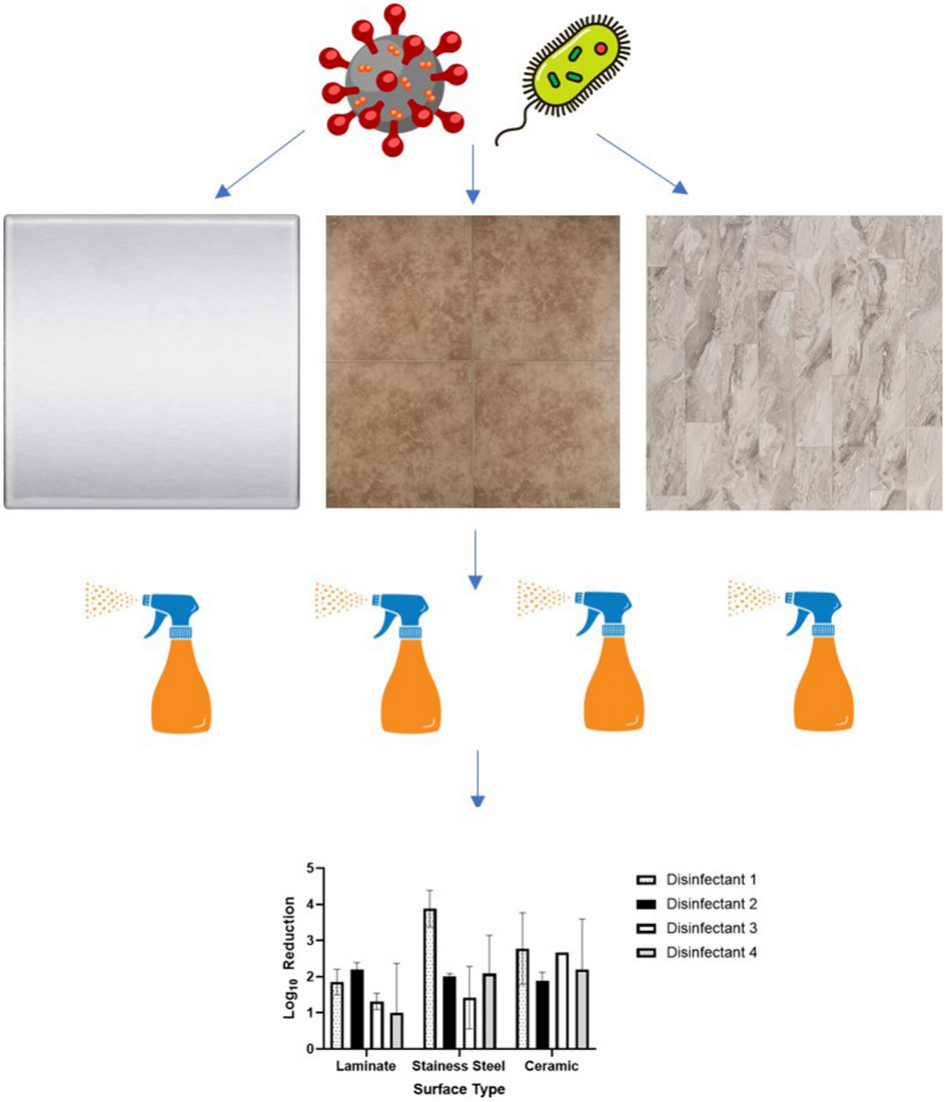


## Introduction

During the 2020 outbreak of Severe Acute Respiratory Syndrome Coronavirus 2 (SARS-CoV-2), the causative agent of COVID-19, contaminated surfaces were proposed as a potential source for the dissemination of viral particles (1). Respiratory viruses are known to survive for hours to days on such surfaces (2–4), and viral nucleic acids have been detected on surfaces in health care, and community settings (4–7). Enhanced environmental cleaning was therefore recommended early in the pandemic as a component of outbreak control for SARS-CoV-2 (8).

As of May 2021, the United States Centers for Disease Control no longer states that SARS-CoV-2 is likely transmitted through surface contamination (8), but is more likely spread through aerosolization of viral particles (9). Despite this, much effort went into the cleaning of environmental surfaces during the pandemic and continues to be a major component of infection control protocols in areas of interaction both in hospital and community settings. Generally, surface disinfection is typically performed through the manual application of liquid or spray disinfectants. Here, we tested the effectiveness of an essential oil disinfectant on SARS-CoV-2 surrogate microorganisms. Phi 6, an RNA pseudomonas phage, has been previously evaluated as a surrogate for multiple virus pathogens including ebolavirus (10–12) and various human respiratory viruses (13, 14), including coronaviruses such as SARS-CoV-2 (3, 15, 16) on surfaces (16–18). We have also examined the use of these disinfectants against a bacterial target, *Staphylococcus aureus*, known to be important in health care and food service settings where the disinfection of pathogens on surfaces is important (19, 20).

Essential oil disinfectants have been proposed for a variety of uses, particularly in the inhibition or inactivation of influenza viruses *in vitro*, with cultured cells (21–26). Benefits of using essential oils as alternative disinfectants include their potential for application on porous surfaces that may not be effectively reached by traditional chemical disinfectants as well as the ability to combine essential oils in blends that affect microbes at different stages in the life cycle. In this study, we evaluate four different blends of essential oils on three different surface types using two SARS-CoV-2 microbial surrogates, a bacterial and a viral surrogate.

## Materials and Methods

### Test Organisms

Bacteriophage phi 6 and host *Pseudomonas syringae* were kindly supplied by the Water Institute laboratory of the Gillings School of Public Health at the University of North Carolina at Chapel Hill. Using prepared 18-h growth of *P. syringae* in 50 ml tryptic soy broth (TSB), bacteriophage phi 6 was propagated by reconstituting with 1 ml of prewarmed (37°C) TSB. Five hundred microliters of reconstituted phi 6 was transferred into 50 ml of fresh TSB with 100 ul of host *P. syringae*, and incubated at 22°C with gentle agitation (100 rpm) for 18 h. The phi 6 stock titer was ~10^9^ plaque forming units (PFU)/ml.

Bacteria *S. aureus* was obtained from the American Type Culture Collection (ATCC 12600) and prepared as previously described (27). The *S. aureus* stock titer was approximately 10^8^ colony forming units (CFU)/ml.

### Essential Oil Disinfectants

Four essential oil blends produced and delivered free of charge by EPS Fragrances (Istanbul, Turkey) were examined. These essential oil blends were designed to target viruses, including SARS-CoV-2 and included a proprietary mix of essential oils at various concentrations (Table 1).

**Table 1:** Components of essential oil mixtures.

**Scientific Name**	**Common Name**
*Melaleuca alternifolia*	Tea Tree
*Eucalyptus globulus*	Blue gum
*Rosmarinus officinalis*	Rosemary
*Curcuma longa*	Turmeric
*Zingeber officinale*	Ginger
*Citrus aurantifoila* Swingle peel	Lime
Cinnamomum zeylanicum	Ceylon cinnamon
*Santalum album*	Sandalwood
*Ormenis mixta*	Moroccan chamomile
Rosa damascena	Damask Rose
*Citrus aurantum* flower	Bitter orange
*Pogostemon cablin*	Patchouli
*Comiphora myrrha*	Myrrh
*Dipterocarpus turbinatus*	Gurjan tree
*Cyperus scraiosus*	Cypril or nutgrass
*Liquidamber styraciflua*	Sweetgum
Pogostone	-
Benzyl benzoate	-
Methyl cyclopentanone	-

Disinfectant 1 (NewAnti FL) was a solution of 5% disinfectant 2 (AntiVir19ED) in 95% ethanol. Disinfectant 2 was undiluted AntiVir19ED. Disinfectant 3 was undiluted Anti-COV19VERS4. Disinfectant 4 was 5% disinfectant 3 (Anti-COV19VERS4) in 95% ethanol.

Disinfectants were provided as either sprays or liquid chemicals and redistributed into 2oz spray bottles for application on surfaces for microbial disinfection.

### Surface Inoculation and Sampling

The effectiveness of the four disinfectants was examined on three different surface material: laminate, stainless steel, and ceramic tile. As the laminate surface was more porous than the other two surface material, it was not possible to decontaminate this surface between disinfectant trials; therefore, a new piece of laminate tile was used for each experiment. The stainless steel and ceramic tiles were cleaned and autoclaved between each use.

For each trial run, microbes were applied to the tile surface and allowed to dry for approximately two min. Then spray disinfectants were applied onto experimental surfaces and allowed to air dry. Cultures were collected from control surfaces before the addition of spray disinfectants. CultureSwabs (Becton Dickinson) premoistened with sterile Dey-Engley neutralizer were used to collect samples from each experimental surface. In addition to experimental surfaces, control surfaces were also sampled during the course of the experiment. Two control surfaces were prepared for each tile surface (1) a negative control surface inoculated with phosphate buffered saline, and (2) a non-spiked surface; both surfaces were sampled using the same methods as the experimental surfaces. Samples of bacteria were detected using a standard bacteriological spread plate method (27) and bacteriophage viruses were cultured using United States Environmental Protection Agency (US EPA) method 1602 (28).

### Statistical Analysis

**Figure 1:**
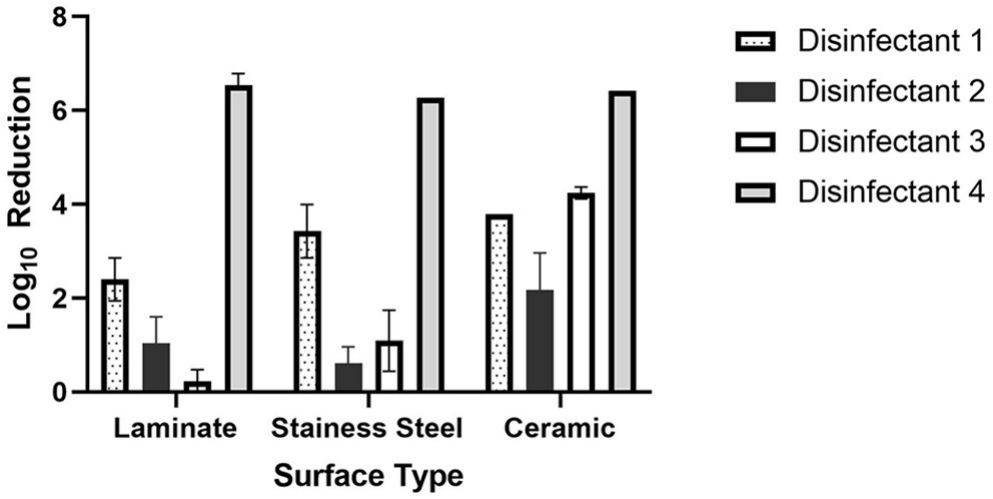
Log_10_ reductions of bacteriophage phi 6 by disinfectant and surface type.

**Figure 2:**
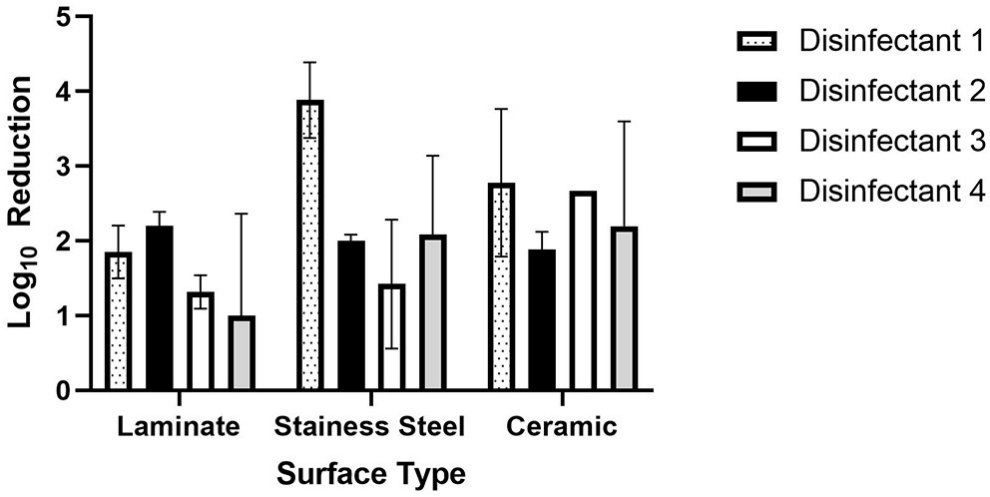
Log_10_ reductions of *S. aureus* by disinfectant and surface type.

Data analysis and graphical representations were created in GraphPad Prism (Version 9). Log_10_ concentration values were calculated by taking the log_10_ of the concentration (N) of each microbe (phi 6 and *S. aureus*) detected at time (t), the end of the experiment, and subtracting the log_10_ concentration of each microbe respectively at time 0. Total log_10_ concentrations for each surface material are shown for phi 6 in Figure 1 and for *Staphylococcus aureus* in Figure 2. A one-way ANOVA (analysis of variance) test was used to determine if there was a difference among mean log_10_ reductions by tile and Tukey's multiple comparisons test was used to examine the relationship between means. All statistical significance was evaluated at an alpha level of 0.05.

## Results

The log_10_ reductions for each microorganism by disinfectant and tile type are summarized in Figures 1, 2. On laminate tiles, disinfectant 1 reduced bacteriophage phi 6 an average of 2.4 log_10_ plaque forming units (PFU) and *S. aureus* 1.5 log_10_ colony forming units (CFU). The log_10_ reduction for disinfectant 2 on this surface for *S. aureus* was also close to two log_10_ (2.2 log_10_ CFU); however, the bacteriophages were decreased to a much lower degree at only 1.0 log_10_ PFU. Disinfectant 3 decreased the *S. aureus* and phi 6 1.3 and 0.2 log_10_ CFU and PFU respectively. On laminate tile, disinfectant 4 was the most effective disinfectant against phi 6 with a log_10_ reduction of 6.5 log_10_ PFU but the least effective against *S. aureus* with a 0.9 log_10_ CFU reduction.

On the stainless-steel surface, disinfectant 1 had the greatest overall effect on *S. aureus* with a log_10_ reduction of 3.9 log_10_ CFU. Disinfectant 1 was also very effective against phi 6 with a reduction of 3.4 log_10_ PFU. Disinfectants 2 and 3 were not very effective against either microbe with log_10_ reductions of 2.0 and 1.4 log_10_ CFU for *S. aureus* and 0.6 and 1.0 log_10_ PFU for phi 6 respectively. In contrast to disinfectant 1, disinfectant 4 was the most effective for phi 6 with a reduction of 4.2 log_10_ PFU but only moderately effective for *S. aureus* with a reduction of 2.2 log_10_ CFU.

On the ceramic surface, the bacterial log_10_ reductions were very similar for all four disinfectants, each falling within the rage of 1.8–2.7 log_10_ CFU (<1 log_10_). For the bacteriophage phi 6, disinfectant 4 was the most effective with a log_10_ reduction of 6.4 log_10_ PFU, followed by disinfectant 3 with a log_10_ reduction of 4.2 log_10_ PFU.

In order to compare the differences between mean log_10_ reduction across surface type by disinfectant, a one-way ANOVA was conducted followed by a Tukey's multiple Comparison Test (Table 2). Based on this analysis, there were no significant differences between disinfectants 1, 2, 3, or 4 on laminate or ceramic surfaces when disinfecting *S. aureus*. However, there was difference in the disinfection of *S. aureus* on stainless steel surfaces between disinfectants 1 and 3, which have average log_10_ reductions of 3.9 and 1.4 log_10_ CFU respectively.

**Table 2:** Tukey's multiple comparison test of the relationship between log_10_ reduction means by disinfectant and surface type.

	**Laminate**		**Stainless Steel**		**Ceramic**	
**Disinfectant**	**Mean Difference**	***P*-Value**	**Mean Difference**	***P*-Value**	**Mean Difference**	***P*-Value**
**Comparison**	**(95% Confidence Interval)**		**(95% Confidence Interval)**		**(95% Confidence Interval)**	
**Phi 6**						
1 vs. 2	1.36 (0.30, 2.41)	0.015^*^	2.82 (1.60, 4.04)	<0.001^*^	1.62 (0.57, 2.67)	0.005^*^
1 vs. 3	2.18 (1.12, 3.23)	0.001^*^	2.33 (1.12, 3.56)	0.001^*^	−0.45 (−1.50, 0.60)	0.54
1 vs. 4	−4.14 (−5.20, −3.08)	<0.001^*^	−2.85 (−4.07, −1.63)	<0.001^*^	−2.63 (−3.68, −1.58)	<0.001^*^
2 vs. 3	0.82 (−0.23, 1.89)	0.14	−0.48 (−1.70, 0.74)	0.61	−2.07 (−3.12, −1.03)	0.001^*^
2 vs. 4	−5.50 (−6.55, −4.44)	<0.001^*^	−5.67 (−6.89, −4.45)	<0.001^*^	−4.25 (−5.30, −3.20)	<0.001^*^
3 vs. 4	−6.32 (−7.38, −5.26)	<0.001^*^	−5.18 (−6.40, −3.97)	<0.001^*^	−2.18 (−3.23, −1.13)	0.001^*^
***S. aureus***						
1 vs. 2	−0.35 (−2.24, 1.54)	0.93	1.88 (−0.02, 3.79)	0.05	0.90 (−1.37, 3.16)	0.61
1 vs. 3	0.54 (−1.35, 2.42)	0.80	2.46 (0.56, 4.37)	0.014^*^	0.11 (−2.16, 2.37)	1.00
1 vs. 4	0.86 (−1.03, 2.74)	0.51	1.80 (−0.10, 3.71)	0.06	0.58 (−1.68, 2.84)	0.84
2 vs. 3	0.89 (−1.00, 2.77)	0.48	0.58 (−1.33, 2.48)	0.77	−0.79 (−3.05, 1.48)	0.69
2 vs. 4	1.21 (−0.68, 3.09)	0.25	−0.08 (−1.99, 1.82)	1.00	−0.32 (−2.58, 1.95)	0.97
3 vs. 4	0.32 (−1.57, 2.21)	0.95	−0.66 (−2.57, 1.24)	0.69	0.47 (−1.79, 2.74)	0.91

* *Significant at the 0.05 level*.

In the comparison of disinfectants used against bacteriophage phi 6, most were statistically significantly different from one another based on mean log_10_ reduction analysis. Disinfectants that were not different include disinfectant 2 and 3 on laminate and stainless-steel surfaces and disinfectants 1 and 3 on ceramic surfaces.

## Discussion

In this study, we found that essential oil disinfectants were able to reduce bacterial and viral microorganisms up to 6 log_10_ PFU for bacteriophage phi 6 and up to 4 log_10_ CFU for *S. aureus*. There was a statistically significant difference by Tukey's Multiple Comparison Test between all disinfectants and disinfectant 4 for phi 6. This result indicates that disinfectant 4 (5% Anti-COV19VERS4 in 95% ethanol) was the most effective for reducing bacteriophage phi 6 on all surfaces considered. For *S. aureus*, there was not one disinfectant that was clearly the most effective. There was a statistically significant difference between disinfectants 1 and 3 on the stainless steel surface, but this is primary due to the high efficacy of disinfectant 1 on this surface. During the coronavirus pandemic, there has been increased attention to surface disinfection methods, particularly in common areas and highly trafficked surfaces. The application of spray disinfectants, such as those examined in this study, provide a rapid and effective means to reduce bacterial and viral contamination on these surfaces.

Previous studies have determined that human coronaviruses can survive on dry surfaces for up to 9 days (8, 10), and that SARS-CoV-2 in particular is viable on plastic and stainless steel for up to 72 h after aerosol contact (11). Despite this, SARS-CoV-2 is readily inactivated by lipid solvents; and multiple studies have evaluated the efficacy of ethanol at various concentrations (29) against viral agents. In suspension tests with ethanol, concentrations of SARS-CoV has been shown to be reduced >5.5 log_10_ tissue culture infectious doses (30). On surfaces, including porcelain and ceramic, spray applications ethanol have shown an inverse relationship between log_10_ reduction and ethanol concentration. A recent study has shown that 95% ethanol was only able to achieve ~2 log_10_ reductions in infectivity (31), indicating that the blend of essential oils examined in this study is more effective in combination with ethanol than the lipid solvent alone. Although surfaces are no longer considered a high risk fomite for COVID-19 transmission (8), the disinfection of microorganisms on surfaces remains an important consideration for high touch areas in hospitals, waiting rooms, etc. Recently detected and continually evolving variants of SARS-CoV-2, such as the delta variant, may have greater transmissibility. As transmission of SARS-CoV-2 variants on surfaces has not yet been examined and it has been proposed the viral load of the SARS-CoV-2 virus in individuals infected with these strains of the virus is higher than with previous variants (32), it will be important to continue with multilayered control measures and surface cleaning procedures. The use of essential oils as disinfectants and the results presented here provide important context in light of the current coronavirus pandemic. As variants continue to emerge, new technologies and methods of delivering disinfectants are important to preventing the spread of pathogens on surfaces and through contact with fomites. As our results show, the use of dilute essential oil blends on surfaces may be an alternative for high concentration lipid solvents in some situations, as we determined similar log_10_ reductions during spray application.

Antimicrobial properties of essential oils have been previously evaluated for both bacteria and viruses (33). Despite this, the mechanism of action of essential oil inactivation or disinfection is not fully understood. Although not the primary focus of our pilot study, previous research has suggested that potential mechanisms for viral inactivation with essential oils may be due to either damage to virus particles and the inhibition of virus adsorption to host cells (33). In work with influenza A, an enveloped virus, authors concluded that essential oils did not prevent adsorption of virus to host cells (23, 34); however, in work with herpes simplex virus, a non-enveloped virus, other researchers proposed that the mechanism of action was direct binding to the virus and inhibition of virus adsorption to the host cells (35–37). In our work with essential oil blends, it is clear that inactivation is occurring on surfaces spiked with microorganisms, but it is outside of the scope of this pilot study to elucidate mechanisms of action for the essential oil blends.

Limitations of our study include the use of a bacteriophage instead of the evaluation of a viral pathogen. However, there is evidence that bacteriophage phi 6 is a reliable model for the survival of coronaviruses under various conditions (38–42). Further research is recommended to include additional viruses, such as SARS-CoV-2 and influenza viruses. A second limitation includes the use of only one type of spray application of these disinfectants. It may be that there are other delivery methods that are more effective in applying or distributing these essential oil disinfectants. Our primary goal was to conduct a pilot study to evaluate the efficacy of the disinfectants on microorganisms, but future work should evaluate the delivery method.

Our results suggest that the application of dilute essential oil disinfectants by using a spray delivery device is an effective way to reduce concentrations of viral and bacterial microorganisms on ceramic, stainless steel, and laminate surfaces. Additional studies are needed to evaluate the utility of these sprays in community settings and to optimize the method of delivery in the decontamination of surfaces.

## Data Availability Statement

The raw data supporting the conclusions of this article will be made available by the authors, without undue reservation.

## Author Contributions

EB wrote the manuscript and conducted the data analysis. MC performed laboratory experiments. HE provided essential oil disinfectants. JB, NB, and AS conceived of the idea and developed the study. All authors have read, reviewed and agreed to the manuscript.

## Funding

Funding for this work was provided by discretionary funds at Whitacre College of Engineering Dean's office at Texas Tech University and was supported by EB's discretionary funding in the Department of Public Health at Texas Tech Health Sciences Center.

## Conflict of Interest

The authors declare that the research was conducted in the absence of any commercial or financial relationships that could be construed as a potential conflict of interest.

## Publisher's Note

All claims expressed in this article are solely those of the authors and do not necessarily represent those of their affiliated organizations, or those of the publisher, the editors and the reviewers. Any product that may be evaluated in this article, or claim that may be made by its manufacturer, is not guaranteed or endorsed by the publisher.

## Abbreviations

ANOVA: analysis of variance
ATCC: American Type Culture Collection
CFU: colony forming units
COVID-19: novel coronavirus 19 disease
PFU: plaque forming units
SARS-CoV-2: Severe Acute Respiratory Syndrome Coronavirus 2
TSB: tryptic soy broth
US EPA: United States Environmental Protection Agency.
